# “Not What I Would Have Pictured Stalking to be or Look Like”: Awareness of Stalking and Views on Stalking Education Among Young People in Wales

**DOI:** 10.1177/08862605261444659

**Published:** 2026-05-23

**Authors:** Sophia Kier-Byfield, Sarah Wallace, Emily Underwood-Lee, Michelle Whelan, Elinor Spiers-Morgan

**Affiliations:** 1 University of South Wales, Pontypridd, UK; 2 Calan DVS, Neath, UK; 3 South Wales Police, Cardiff UK

**Keywords:** stalking, youth violence, adolescents, bullying, violence prevention

## Abstract

Young people represent the highest risk group for stalking in England and Wales, yet research on young people’s stalking behaviours and perceptions is scarce. The research available about young people’s baseline comprehension of stalking suggests widespread misunderstandings and views that could minimise responses to concerning behaviours. The project *Stalking and Young People in Wales: Exploring and Increasing Knowledge, Awareness and Understanding* sought to gain an insight into the knowledge and awareness of stalking among young people aged 16 to 24 in Wales. Through eight participatory workshops across Wales, the team engaged with 84 young people to learn more about their perceptions of stalking, levels of confidence regarding terminology and the law, and their views on stalking education, including what young people need to know and how they want to receive information. Workshop findings suggest that although stalking knowledge exists, it is often limited and supported by stalker stereotypes that do not consider the high likelihood of stalkers being acquaintances. There was also evidence of confusion around terminology and what might constitute stalking, as well as limited experiences of stalking education. Young people expressed a desire for more information and education. This article presents an overview of the project findings, along with suggestions for practice and future research.

## Introduction

Recent figures suggest that “around one in seven people aged 16 years and over in England and Wales has been a victim of stalking at least once” (Office for National Statistics, 2024). The data suggest that the likelihood of being a victim of stalking was higher for young people, particularly young women in the age bracket 16–19 (Office for National Statistics, 2024). These figures are likely to be underestimated, as stalking can be underreported or misunderstood by those impacted, authorities and wider society (Brady & Nobles, 2015; Suzy Lamplugh Trust, 2023a). The impacts of stalking can be immense in relation to mental and physical health, education and long-term well-being (Purcell, 2009).

Despite growing recognition of stalking as a serious offence, there remains limited research on how young people perceive, identify and respond to stalking behaviours in their everyday lives. This gap is significant, as without establishing a concrete baseline of young people’s awareness that is based on a consistent definition of stalking, findings about prevalence may be skewed or incomplete (Cloonan-Thomas et al., 2021). Recent research carried out in various parts of the UK suggests stalking is common among young people yet difficult for them to identify, that they are vulnerable to specific risks connected to their age and use of technology, and that their concerns are not always met with informed responses (Cater, 2025; Mclean et al., 2025). Stalking perceptions may also vary based on context and culture. The project *Stalking and Young People in Wales: Exploring and Increasing Knowledge, Awareness and Understanding* (from here on *Stalking and Young People in Wales*) engaged with young people aged 16 to 24 through workshops across Wales to explore stalking. This article presents findings from the workshops, their implications and recommendations for further research.

### Defining Stalking

Stalking is recognised as part of the broad spectrum of Violence Against Women, Domestic Abuse and Sexual Violence (VAWDASV) in Wales and Violence Against Women and Girls in England (UK Government, 2021; Welsh Government, 2022). However, stalking can also happen outside of the paradigms of gender-based and intimate partner violence. A complex crime, stalking is not legally defined in the United Kingdom. It is generally understood to be a “Fixated, Obsessive, Unwanted and Repetitive” course of conduct (“FOUR”; Home Office, 2024), although this conceptualisation is sometimes critiqued for relying on subjective views of fixated/obsessive (Independent Office for Police Conduct, n.d). Stalking can comprise “following, contacting, publishing material relating to the victim, monitoring, loitering, interfering with property and watching or spying,” but behaviour lists are never finite (Suzy Lamplugh Trust, n.d.). Legally, serious stalking “causes fear of violence or serious alarm or distress” (Alice Ruggles Trust, n.d.), setting it apart from harassment. Serious alarm and distress are also not defined in the U.K. legal context but could involve victims having to “suffer emotional or psychological trauma or [having] to change the way they live their life” (Suzy Lamplugh Trust, n.d.). The difficulty in defining stalking reflects the complicated nature of the crime, despite its prevalence and the severity of its impacts (Storey et al., 2023). The Stalking Super Complaint, submitted by the Suzy Lamplugh Trust (2022) on behalf of the National Stalking Consortium, brought further attention to the need for an improved police response to reports of stalking and.At the time of writing, a legal review is underway (Home Office & Philips, 2025), and organisations such as the Crown Prosecution Service are committing to improving their understanding of stalking (Crown Prosecution Service, 2026). Arrest rates, prosecutions and convictions remain low, and there are challenges in reporting and gathering evidence (Mayor’s Office for Policing and Crime, 2024; Taylor-Dunn et al., 2018). The true number of cases may not be reflected within official data, indicating a “dark figure” of stalking (Brady & Nobles, 2015, p. 3165).

### Stalking Prevalence Among Young People

Barr and Newman’s (2024, p. 63) review of the adolescent stalking literature suggested “a prevalence of between 5.3% and 9% and up to 36% of young people if a wider definition of stalking in which one stalking behaviour is included,” rather than the usual two that are required as minimum for reporting. Research in London has found that “the victim age group with the largest proportion of [serious] offences was under 18 (39% *n* = 88), followed by 18 to 24-year-olds (34% *n* = 451)” (Mayor’s Office for Policing and Crime, 2024), which may suggest more severe crimes in this age group or escalation as a result of delayed recognition or reporting. Rates of cyberstalking among young people are also reported to be high (Brown et al., 2021; Smith-Darden et al., 2017). According to the Suzy Lamplugh Trust (2023b, p.1), “between March 2022 and February 2023, 1,182 young people aged 25 and under reached out to the National Stalking Helpline, including 150 young people aged 18 and under.” Studies that report stalking experiences specifically are crucial, as stalking can be omitted from surveys on young people’s experiences of violence (Youth Endowment Fund, 2024).

### Stalking Behaviours and Perceptions Among Young People

Roberts et al.’s (2016) review of stalking among 13 to 17 year olds found some differences in behaviours to adults, such as a greater prevalence of same-gender stalking among girls, stalking relating to bullying and complications when it comes to group or solitary intimidation; a greater likelihood of being stalked by an acquaintance than a former romantic partner; the stalking of adults by young people. As Worthington (2023) has argued, there is a bias towards expecting stalking to be romantically motivated that may need to be challenged when considering young people, especially when some research suggests a high rate of stalking associated with bullying and deteriorating friendships, with lower rates of stalking being reported as involving ex-partners (Pereira & Matos, 2016; Purcell et al. 2009).

The review by Roberts et al (2016) was updated by Barr and Newman (2024) to consider more recent research, expand search terms and include children. Their review adds further complications to the available picture of stalking among young people, as they note considerations such as younger victims not describing a brief period of intimacy as a romantic partner; cases of young people stalking adults being considered as more severe; reliance on court data in figures only representing cases that have made it through the criminal justice system, which is a low percentage of cases; a lack of clear evidence of the impact of ethnicity, sexuality and gender-identity; a lack of understanding of how bullying dynamics link with stalking. The phenomenon of young people self-reporting being both a victim and perpetrator of stalking further complicates the picture (Cloonan-Thomas et al. 2022; Rothman et al., 2021), in addition to research that highlights only a small number of cases with reported sinister intent (Ybarra et al., 2017). It has therefore been concluded that helping young people to recognise unacceptable behaviours would be an effective first step (Barr & Newman, 2024). Furthermore, misguided or biased perceptions exist among young people, such as the assumption that stalking is more serious when perpetrated by a stranger (Chung & Sheridan, 2022; Scott & Sheridan, 2011), and a general minimisation of criminality, for example, viewing cyber stalking as less serious (Janickyj et al., 2025). Perceptions may also vary by location (Chung & Sheridan, 2022), highlighting the importance of context-specific research. Wales, a devolved nation within the United Kingdom with a population of just over 3 million, is the context in which the *Stalking and Young People in Wales* project took place, although the article may offer transferable findings across United Kingdom and international contexts.

## The *Stalking and Young People in Wales* Project

The project was led by researchers at the University of South Wales in partnership with frontline service Calan DVS and South Wales Police. Funding was secured from the Vision Small Projects Fund and the UK Prevention Research Partnership. Connections, support and expertise were also enhanced through an advisory group, which involved representatives from the Offices of the Police and Crime Commissioners in the different regions of Wales, stalking survivors and researchers. The research questions were as follows: (1) how confident are young people in their understanding of stalking?; (2) what misunderstandings of stalking might young people in Wales have?; (3) what education have they either had or would they like to see in future?

### Methods

In-person workshops were the primary method of qualitative data generation. While the data collection remained researcher-led, the workshop structure allowed young people to engage as active contributors. The workshops comprised several sequential activities that contained a mix of individual and group work and involved moving around the room where possible (Table 1). Activities 4 and 5 were inspired by the Nominal Group Technique: small groups were asked to brainstorm their priorities before choosing their top five. During a break, the top five from each group were combined by the facilitators to create an overall list without duplicates. Each participant could vote up to five times for any priority on the overall list, including using all their votes on one priority or spreading them across. The final activity was a short survey to capture further opinions and feedback. Workshops were designed with the principles of participatory research in mind (Vaughn & Jacquez, 2020), positioning young people as experts in their experience and knowledge as collaboratively generated. However, due to the restrictions of a one-year project, young people were only engaged during the workshops. There were therefore inevitable power dynamics with this approach despite its workshop-based format (Wilkinson & Wilkinson, 2017). For example, we were aware that the workshop environment could still represent a traditional learning environment for some, as it was facilitated by researchers/educators and involved a set of activities with instructions and a pre-determined structure. Workshop plans were discussed with our advisory group and experts from Paladin National Stalking Advocacy Service, and extensive feedback was integrated into the plan.

**Table 1.** Workshop Format.

*Welcome and project overview*	• Introductions • Content warning • Workshop purpose • Consent • Group agreement: Chatham House Rule
*Activity 1 – Individual Brainstorm*	• Complete the following sentence as many times as you like on post-it notes (one response per post it, drawings welcome): *When I hear the word/think about stalking, I think about*. . .
*Activity 2 – Agree/Disagree/Not sure*	• Move to a table to express whether you *Agree*, *Disagree* or are *Not Sure* about the following statements: ○ I understand what “stalking” means and involves ○ I understand what “harassment” means and involves ○ Stalking is a crime ○ I understand the difference between acceptable and unacceptable behaviours ○ I would speak to someone or get help if I was using/experiencing stalking ○ I know where to go to get support for stalking behaviours ○ I know where I would go to report stalking
*Activity 3: Discussion*	• Discussion prompt: “There is no legal definition of stalking in the UK, but it is generally agreed to be ‘a pattern of unwanted, fixated and obsessive behaviour which is intrusive and causes fear of violence or serious alarm or distress.’” (Alice Ruggles Trust, n.d.) • Discussion prompt: Video intended for young people about stalking (Derbyshire Constabulary, 2022)
*Activity 4: Group Priority Setting and Ranking*	• In your group, discuss what is most important for young people to increase their knowledge, awareness and understanding of stalking • Decide on the top 5 priorities with your group • Each group’s top 5 priorities will be summarised, which you will then be invited to individually rank (5 votes per person), resulting in a whole group list of top priorities.
*Activity 5: Actioning priorities*	Discuss factors for actioning your priority, for example, type of format/learning, accessibility. Actions will be summarised and ranked before.
*Post-workshop survey*	Web and paper-based survey capturing anything missed in the workshop, further suggestions, and information on prior stalking education.

### Workshop Recruitment

The project aimed to engage 120 to 150 young people aged 16 to 24 through in-person workshops of up to 30 young people, together with two online workshops. However, plans were adapted to better suit young people, with the team travelling to the places where they usually spend time. The revised approach comprised eight smaller, in-person workshops within education settings and youth clubs. All workshops were facilitated by the first and second author with support from the groups’ responsible adults, delivered between February and April 2025.

### Ethical Considerations

High-risk ethics approval was secured via the University of South Wales Faculty Ethics Sub-Group. Organisations were provided with a leaflet, a Participant Information Sheet, and the study Consent Form to discuss with young people. Recruitment was led by the gatekeeper organisations. It was made clear to young people that they would not be asked about any personal experiences of stalking and that the Chatham House Rule would be in effect (Chatham House, n.d.), in addition to anonymous data collection (post-it notes, flip chart paper and survey). A £20 voucher was offered to each participant, and food was provided. The team considered the debates surrounding compensation/incentives for young people in research (Afkinich & Blachman-Demner, 2020), but it was decided in consultation with the advisory group that the offering was appropriate for the age group and necessary for the three-hour time commitment.

### Analysis

The first and second authors met face-to-face on several occasions to organise the data (post it notes, ranked priorities, discussion notes). The responses from Activities 1 and 3 were entered into an Excel spreadsheet and grouped thematically with any relevant quantitative aspects (e.g. number of responses per question) counted and recorded. For Activity 2, the categories of *Agree*, *Not Sure* or *Disagree* pre-determined how responses were grouped. For Activity 4, the top priorities from each workshop ranking exercise were recorded with the number of correlating votes, before being combined into a further eight over-arching priorities representative of the votes. The ideas generated during group discussions in Activity 5 were approached in a similar way, with each statement being recorded and grouped, although items were not compressed.

The overall approach to the analysis takes inspiration from reflexive thematic analysis (RTA), and we have aimed to be “knowing” in our approach (Braun & Clarke, 2022). As team members, facilitators and those responsible for making sense of the data, the authors were deeply involved in generating the data and telling a story about young people’s perceptions of stalking. For example, Activity 3 had a group discussion format that was led by the facilitators. Rather than audio recording, the co-facilitators wrote down notes from the discussion. As such, the subsequent collaborative efforts to sort the data were not undertaken to ensure reliability of themes, but to inform an iterative and discussion-based process that honoured the intense, productive and messy nature of the workshops. We actively recognise that the planned activities and questions would also have been influenced by our interests and pre-existing ideas of what young people might know or not know, which in turn are based on personal experiences (survivors and experts in the advisory group; team members’ personal experiences; team members’ children) and encounters with the existing literature. It would be easier to tell an anticipated story about young people simply not understanding stalking, but young people should not be underestimated. We strive to present a nuanced picture.

We diverge from RTA by presenting some quantitative representations for Activities 1, 2, 4 and 5. This provides a bigger picture of the cohort (*n* = 84) and communicates potential patterns and prominence within limited space. However, responses detailed numerically are not directly correlated to the number of participants, as some engaged more actively than others in each workshop, and overall participation varied. Furthermore, in Activity 1, participants were encouraged to answer as many times as they wanted, and in Activity 2, it was not possible to cover all statements with each group due to time constraints. In some instances, the activities were not completed if groups were less engaged, or if the previous discussions lasted longer. Therefore, any numerical representations are not intended to reduce the findings to a quantifiable, positivist representation, but to add to the complex picture of stalking perceptions that we are curious about and that warrant further investigation beyond the scope of this paper. What we present here is incomplete and partial, but nevertheless valuable.

## Findings

The team travelled to 6 areas and 84 young people engaged across 8 workshops (Table 2).

**Table 2.** Workshop Locations and Participant Numbers.

Location	*n*
Cardiff (3 separate sites)	48
Neath Port Talbot	2
Newport	7
Brecon	15
Rhyl	6
Haverfordwest	6
*Total*	*84*

### Activity 1 – “When I Think About/Hear the Word Stalking, I Think About. . .”

The first activity was carried out individually: young people were encouraged not to talk to or look at other participants’ responses to best capture their existing perceptions, although this was not always avoidable. Responses were subsequently grouped into 7 over-arching themes, supported by 19 subthemes (Table 3).

**Table 3.** Over-Arching Themes From Activity 1.

Main theme	Sub-theme	Frequency of post it notes
Perceptions of how stalking is perpetrated/behaviours	• Tech-facilitated stalking • Physical stalking • Both online and physical • Potential outcomes	185
Why stalking takes place	• Potential positive reasons • Negative reasons	6
Perceptions of the stalker and who is stalked	• Stalker gender • Stalker profile • Stalker traits • At risk groups • Interpersonal dynamics	48
Representations of stalking	• TV and Film • Novels • Harmful representations	33
Perpetration – when and where	• Time of day • Locations	12
Emotional responses	• What stalking might feel like • Opinions/feelings about stalking	81
Support	• Routes to and lack thereof	4

There was a breadth of in-person and online behaviours highlighted. 53 responses detailed tech-facilitated approaches to stalking, for example “online,” “social media,” “looking at/watching someone’s social media,” “making fake accounts,” “taking photos without consent,” “surveillance”; 77 responses detailed physical behaviours, for example “following,” “watching,” “keeping an eye on where that person lives”; 32 responses detailed approaches that could be both cyber or physical stalking, for example “unwanted attention”; “sending unwanted gifts”; “unwanted communication”; “not leaving someone alone”; “finding information about a person without speaking to them.” Although there were a few miscellaneous or unusual responses (such as “noticed” or “marked”) and indications of clear confusion between stalking and other potential pursuit behaviours (“Pros for stalking–good stalking–what if I am looking for family?”), the majority were consistent with stalking behaviours.

However, there are some notable trends in the data from Activity 1 warranting further attention. First, “following” in the physical stalking subtheme was most frequently referred to, with 50 responses. It was also suggested that stalking mostly takes place at “nighttime” and in places such as “dark alleys” or “bus stations.” Whilst physical following is certainly a common stalking behaviour (Storey et al., 2023; Suzy Lamplugh Trust, 2023b), a potential misunderstanding could lie in the idea of who would be following a person. For instance, deviance was highly prevalent in the *Stalker traits* subtheme. Of 24 responses in this subtheme, only four were about “obsession” and one about “jealousy,” with the remainder reflecting perceptions about stalkers being “depraved,” “dodgy,” “creepy,” a “freak,” or a “druggie.” The *Stalker profile* subtheme descriptors followed a similar pattern, with perpetrators being classified as “sex offenders,” “peado,” or someone who wears a “black hoodie.” The *Stalker gender* in the eyes of young people was always an older male preying on younger girls. These descriptors connect to “peeking” and “peeking through a hole” statements within the *Physical stalking* category. In this activity, only two statements indicated awareness about stalkers potentially being known: “relationships” and “Activities/sport (people you know).”

TV shows and films were mentioned by the participants suggesting that fictional media representations of stalking influence young people’s understanding. Only one statement was about support options (“Police intervention”), whilst other three statements in this subtheme subtheme were more negative (“Little knowledge taught to people,” “lack of support,” “Not knowing what to do”), suggesting that the representation of stalking in the media is more prominent or immediate than knowledge of what can be done to get help. Simultaneously, the young people we spoke to were very aware of the potential emotional impact of stalking, and that it could make someone “scared,” “paranoid,” have “anxiety” and be “life ruining.”

### Activity 2 – Agree/Disagree/Not Sure

Activity 2 asked young people responding to a series of statements through the act of moving to the designated “Agree,” “Disagree” or “Not sure” table and registering their vote (Table 4).

**Table 4.** Responses to *Agree*/*Disagree*/*Not Sure* Activity.

No.	Statement	*Agree*	*Disagree*	*Not sure*
1	I understand what “stalking” means and involves	39	1	29
2	I understand what “harassment” means and involves	34	0	36
3	Stalking is a crime	24	1	12
4	I understand the difference between acceptable and unacceptable behaviours	32	1	10
5	I would speak to someone or get help if I was using or experiencing stalking	18	11	18
6	I know where to go to get support for stalking behaviours	3	2	3
7	I know where I would go to report stalking	25	4	6

Participants were also asked to write down their reason for choosing either *Agree*, *Disagree*, or *Not sure*. Findings indicate that most young people seemed confident that they knew what stalking is: 57% stated they understood, 42% were not sure, and only 0.69% (1 person) said that they disagreed: “Don’t know.” It was clear that some of the young people had a good fundamental understanding: “Stalking is receiving unwanted attention from someone that you either know or don’t”; “An invasion of privacy – it also depends on the user themselves”; “I agree as I believe I have a pretty clear understanding of what stalking entails, for example, traditional following, going to house, sending stuff, modern + social media misuse.” However, there were also unclear responses from those who expressed confidence in their understanding by choosing “Agree”: “I agree, it is a pretty straightforward concept. If someone is following you or separating you from a vast crowd of people with intent to keep it a secret, then I would class that as stalking.” The mention of stalking being a secret here potentially supports some of the stereotypes in Activity 1, which suggests that in some cases confidence might be based on only partial understandings of stalking.

On the statement of knowing what harassment is, young people were slightly less sure but still reasonably confident overall, with 49% of responses stating “Agree” and 51% stating that they were “not sure.” Some young people expressed coherent understandings of harassment, at least to the degree that could captured be in a short exercise/post it note: “Harassment is when someone persists in following you on social media or in person won’t leave you alone”; “Unwanted communication or attention”; “[. . .] harassment is when someone is bothering you and making you feel disturbed and uncomfortable.” Of interest in the latter response is the mention of “disturbed and uncomfortable,” a distinction that potentially sets harassment apart from more serious stalking offences that require fear of violence. However, misunderstandings were also present: “[. . .] stalking is often silent while harassment is verbal.” As noted above, the notion that stalking is always more secretive, silent, or unseen needs to be challenged.

Linked to these findings were responses regarding whether stalking is a crime. Several responses were clearly unsure, which indicates a definitive need for further education and clarity on behaviours, legal thresholds and categorisations. Some young people indicated a deeper understanding of when and why stalking is a crime: “Stalking is a crime, yet it is only after two or more well documented incidents that police get involved.” However, some of the responses from participants who agreed that they knew stalking is a crime suggested that they may be unsure when it becomes a crime, or as above, that there is a limited view of stalking carried out by someone unknown and unseen: “Stalking is a crime because its observing someone from a distance without their knowledge”; “To a certain extent – depends on the situation”; “Yes I agree to a certain extent but it depends on what the situation is”; “Depends on the person’s intentions. Stalking can be worded for many different situations and can be viewed very differently.”

Taken together, these findings suggest that greater knowledge and understanding is needed for young people about both stalking and harassment. Further demonstrating the need for both more information and trust in help seeking is that 38% were unsure if they would speak to someone to get help for stalking, with 23% saying that they would not: “No I wouldn’t, especially if in a place of work or school as worried of being judged or losing my job. I would be worried stalking would progress if I told someone”; “I don’t think I would as I know so many people it’s happened to – they report it or tell someone and nothing comes of it so what would the point be.” Although 71% said they would speak to people or services such as “police,” “college tutor,” or “parent” to report stalking, 11% said that they did not know where to go to report stalking and 17% were not sure: “We’d get judged by fellow people if we went [to] someone; abusive”; “I wouldn’t tell anyone due to trust issues”; “I would report it to my old man because I have had negative actions taken by the police – they are useless.” Some perspectives suggested a deep mistrust in services that could prevent young people from getting help: “I disagree, because although it is a crime, it’s never taken seriously enough. Most of the time you get called dramatic or the problem is minimised. It’s never taken seriously enough until is causes someone’s death, and then it’s too late to take it seriously.”

### Activity 3 – Group Discussion: Stalking Definition and Watching an Example Video

Young people were asked to respond to two prompts: the absence of a legal definition of stalking and a commonly used description of stalking from the Alice Ruggles Trust website (Alice Ruggles Trust, n.d.; see Table 1), and a video intended for young people’s stalking education (Derbyshire Constabulary, 2022; Table 5).

**Table 5.** Activity 3 Themes.

Discussion topic	Themes
Stalking definition (Alice Ruggles Trust, n.d.)	Clear wording
	Lack of nuance
	Desire for a legal definition
	Young people’s language needs
	Young people understanding behaviours
Stalking awareness video (Derbyshire Constabulary, 2022)	Challenging perceptions
	Concern about VAWDASV
	Desire for education
	Duration of video
	Good/clear
	Inclusivity
	Need for breadth of experiences
	Opinions on perpetrator

*Note.* VAWDASV = Violence Against Women, Domestic Abuse and Sexual Violence.

Although some felt the description’s wording was clear and “captures what stalking is,” young people generally felt there was a lack of nuance in the terminology used: “[The] definition doesn’t capture the complexity and breadth of online risks, e.g. dating apps, cat fishing, location updates, watching stories, seeing what you've liked and watched”; “Too loose.” Young people discussed feeling confused about stalking being a crime with no UK legal definition, and that more emphasis on a legal definition might challenge casual references to stalking in their everyday lives: “Stalking is used casually in conversation, e.g. in jest, Facebook stalker”; “When you hear the word abuse, it is taken seriously, but stalking is harder to define/isn’t tangible and could be minimised by young people.” These responses suggest that more care may be needed when explaining stalking to young people, and that relying on general language used in professional or legal contexts might be insufficient to challenge the daily usage and misconceptions about stalking. Matters of language may be particularly important when thinking about inclusive learning: “Quite wordy–some complex wording such as obsessive and intrusive especially for [Additional Learning Needs]/neurodivergent individuals”; “How terms are categorised for e.g. neurodivergent people [matters] as they can take things literally.” Activity 3 findings also suggest that defining stalking for understanding and awareness raising with young people may pose challenges if words such as “obsessive” are relied upon, as “the person being obsessive [might not] realise they are being obsessive.” Furthermore, it was noted that things “can be blurry,” because “if the victim is not scared and being threatened, they might not recognise stalking.”

The video used in the workshop was produced by Derbyshire Constabulary (2022) and portrays a young white woman being stalked by a young white man in her school through a range of physical and online behaviours. It was found through a YouTube search for stalking information videos. The video has no voice-over or dialogue and ends without a resolution. Later discussions with the Youth Engagement Officer from Derbyshire Constabulary revealed that the video available online is a trailer clip of a longer video intended only for use in the force’s facilitated stalking lessons, which does end with the girl contacting the police (Personal communication, Derbyshire Constabulary Youth Engagement Officer, September 4, 2025). However, the shorter version of the video is easily accessible online and could be accessed by young people or used by other organisations for teaching, which the team determined to be the case in Wales (Personal communications, Stori Cymru Spectrum health relationships programme, September 5, 2025). Positively, the young people in the workshops expressed that a video such as this was relevant: “Texting, walking past and filming are all relevant to what we think stalking is.” Some young people wished the video was longer and more in-depth, but showing even the publically available short version did seem to challenge some young people’s perceptions of stalking. This was particularly the case for recognising the risk of familiar stalkers and the less violent behaviours across the stalking spectrum that can still cause distress: “Expected it to be more serious/harmful such as breaking in and following”; “Not what I would have pictured stalking to be or look like”; “Didn’t think stalking would be from someone I know”; “[. . .] might think it is not stalking if it is a person I see in my class everyday.” There were also a variety of opinions on the perpetrator: “The guy believes it is romantic–may not recognise the behaviours as stalking”; “He isn’t aware it is stalking per se but is aware she has asked him to stop”; “Maybe he doesn’t know how to show his feelings.” This feedback suggests it may be particularly important to educate young people that stalkers can be familiar, that stalking can emerge from situations where there are initial feelings or curiosity, and that from a criminal perspective on stalking, intent matters.

Young people expressed an urgent desire for more education: “There should be a full day in school and more often, like e.g. sex education is only one lesson people need more, people can forget, it needs to be repeated”; “Videos only work if you are talking about it too–facilitated discussion is important.” There were feelings expressed that this was extremely important considering the current climate of increased misogyny: “People like Andrew Tate amplify the toxic masculinity, e.g. a no actually means yes.” However, young people also highlighted a need for stalking education to be inclusive regarding representation but also in language and delivery for those who might be neurodivergent: “Should show different perspectives, e.g. boys being stalked, different genders, etc.”; “Reading facial expressions–what if you couldn't”; “Picking up the teddy bear might be seen as accepting the gesture rather than being polite”; “Should include impact on young people, e.g. 13–14 years.” Furthermore, although we used a fictionalised information video as our example, young people expressed an interest in hearing more real stories: “If you are going to educate kids, hearing from survivors about real life experiences is important”; “We need real stories about how it can impact someone and the support they got.”

### Activity 4 – Priority Setting

Whilst young people expressed an initial confidence in their understanding of stalking (Activity 2), Activity 3 portrays a desire for education and opinions among young people about what that education should contain. This was consolidated in Activity 4, where the most highly ranked priority for young people was more education (Table 6).

**Table 6.** Top Priorities.

Priorities	Votes
More education about what stalking is, how to recognise signs, how it differs to other harmful behaviours and staying safe online	122
More education about where and how to get support/report stalking	39
Policy and governance – protocols, processes, support specialists in schools and workplaces, funding	21
More awareness about healthy relationships, consent, boundaries, and respect	19
Making sure education and awareness is accessible and available in different formats	19
Influence of gender and gender expectations on behaviours and perpetuating harms	18
Understanding the law and the legal consequences of stalking	17
More accountability for tech companies	11

### Activity 5 – How to Action Priorities

Young people had a range of ideas about how best to activate their priorities (Table 7). There was an interest in both online/digital interventions and face-to-face learning; follow-ups and making awareness-raising literature widely available were also highlighted.

**Table 7.** Ways to Action the Priorities.

Format	Example ideas	Count
Online/web-based	Informative and digestible videos–for example, showing different perspectives, situations, etc.	35
	Social media campaigns, for example, utilising content creators	
	Social media reels/ads	
	Websites	
	Real-life scenarios, situations, quizzes and games	
Face-to-face/physical	Teaching with group interactions	21
	Social action, for example, creating community art to raise awareness	
	Teaching it in an accessible way – not PowerPoints/blocks of text	
	Physical support spaces where young people can access outside of work/education	
	Separate stalking education in both formal and informal education settings, for example, assemblies, training	
	Include stalking into the wider VAWDASV spectrum in education	
	Provision of school resources – for example, support groups, school notice boards	
Written and illustrated resources	Illustrated resource booklet – not too much writing and accessible	13
	Posters/physical presence of information	
	Develop a toolkit to help professional support young people with stalking concerns	
	Use different languages	

*Note.* VAWDASV = Violence Against Women, Domestic Abuse and Sexual Violence.

### Post-Workshop Survey

Following each workshop, young people were invited to share any final thoughts and feedback. Responses indicate that, for some young people, this had been an opportunity to learn, as 11 young people stated they had not received or accessed any information about stalking (question 5). Additionally, there were five “N/A” and two uncompleted responses to this question, suggesting that from a possible 61 responses, 18 (30%) may have received no prior education on stalking. Where young people had previously received or accessed stalking information, responses indicated varying quality and depth (See question 5, Table 8).

**Table 8.** Post-Workshop Survey Response Examples.

Question and responses received (‘NA’, ‘No’ and ‘nothing to add’ responses removed)	Response examples
1. Which topics or aspects of the workshop did you find most interesting or useful? (*n* = 61)	• *All the topics discussed was great and informative especially the one about the definition of what stalking is.* • *Finding out what stalking actually is and that its illegal.* • *Finding out that stalking can be little things and harassment is different.* • *Hearing everyone else’s insights on staking from different points of view.*
2. Anything that might have been missing from workshop discussions? If so, what? (*n* = 26)	• *I feel a higher priority on digital and online stalking would be good as for young people that's where most of it occurs.* • *More educational parts (even like a booklet with some info about stalking would be really useful).* • *It would be great to make a full day of the workshop.*
3. Further suggestions about how best we can raise awareness of stalking among young people in Wales? (*n* = 55)	• *Videos on stalking showed to kids.* • *Creating more fun media to engage with youth.* • *Do this [workshop] around Wales, bring it to schools.* • *Youth workers can help and talk to students in school.*
4. Further suggestions about how to ensure professionals understand and support young people experiencing stalking? (*n* = 46)	• *Handbooks and workshops and training sessions.* • *Integrating it into youth work and teacher training.* • *1-2-1 conversation and more long-term individual work with a young person to build relationships and trust.* • *Maybe allow these professionals to meet a group of young people to understand the ideas and advice firsthand.*
5. Have you previously received or accessed any information about stalking and where from? (*n* = 42)	• *I was probably taught about it in school, but I honestly can't remember.* • *Only vaguely through movies/books/media.* • *Youth worker at school.* • *Posters in town toilets, leaflets.*
6. Anything else you want to add from the workshop discussions today? (*n* = 14)	• *Society and their impact on stalking should be talked about and studied more.* • *The class and event has been great as I have been warned more about stalking now that I did before we started.* • *Everything I can think of has been covered and I am personally grateful for learning so many things I didn't know about stalking.*

Follow-up feedback sessions were also originally planned with young people to consolidate emerging resources that the project team had pledged as part of the project. Due to a time-limited one-year project and the clash with summer holidays in 2025, none of the young people were reachable. At the time of writing, the resource-creation is ongoing with two awareness-raising videos being co-produced with media students at University of South Wales and Cardiff Metropolitan University. The team aims to get feedback on the resources from young people.

## Discussion

This project aimed to explore (1) young people’s confidence in their understanding of stalking; (2) what misunderstandings of stalking young people in Wales might have; and (3) what they would like to see in stalking education. Encouragingly, findings suggest an awareness of the dangers and a condemnation of stalking. However, also evident was that young people’s awareness of who a stalker might be and their motivations might be biased towards stalker tropes, with less awareness of the dangers and signs of known and visible stalkers. This could in turn lead to an inability to recognise everyday behaviours associated with stalking, such as fixated behaviours concerning familiar people, particularly when these tropes are identifiable in some support resources (Meic Cymru, n.d.). It was also promising to see an awareness of the importance of the unwanted nature of stalking behaviours, and there were informed responses from young people that related to finding information, help seeking and contacting family, friends and authorities. However, knowledge and confidence were also mixed, with more clarity needed for understanding stalking, harassment and the difference between the two, and greater awareness of and trust in help-seeking. Our findings reflect those of other studies, which suggest that young people may minimise stalking (Chung & Sheridan, 2022; Janickyj et al., 2025; Scott & Sheridan, 2010).

Young people expressed particular concern over the lack of a stalking definition, especially when paired with the fact that both within daily life and in the media, the term is used to refer to a range of non-threatening behaviours, such as checking out peers or love interests on social media, spending a lot of time with someone or bumping into someone often. Everyday use of stalking terminology has been shown to be common among young people (Howard et al., 2019; Spitzberg, 2017; McLean et al., 2025). We asked young people about their understanding of stalking and harassment because they are often grouped or used interchangeably in ways that reflect the legal positioning of stalking as an obsessive form of harassment (Police.uk, n.d.). Whilst there are overlaps and similarities in behaviours, grouping stalking and harassment together can create confusion about the signs of severity for both those impacted and the authorities, which can lead to detrimental outcomes for victims (Bracewell et al., 2020).

In research on higher education contexts, for example, the emphasis on sexual violence and harassment may be obscuring the collection of accurate data on specific abuses such as stalking (Bull & Bradley, 2023). The collection of accurate data is complicated when dissonance between the tools available and the varying definitions are considered (Bull & Bradley, 2023), in addition to a lack of information about young people’s baseline perceptions of stalking. Accurate representations of the harms young people experience in contexts such as higher education are essential considering the heightened risk: “full-time students (7.7%) are the most likely to experience domestic abuse compared to any other occupation,” with women being doubly at risk (Khan 2021). Undergraduate students’ circumstances, such as living in rented accommodation and away from support, overlap with vulnerabilities and risk factors for stalking (Drouet & Gerrard-Abbott, 2023; Roberts et al., 2016). Further complications are added when the prevalence of cyberstalking is considered alongside the ever-changing nature of digital media platforms and the high rates of usage and incidents among young people (Banks & Andersson, 2023; Suzy Lamplugh Trust, 2023b). Clear information and education are needed to raise awareness on the complexities of stalking both online and offline in addition to how stalking links to other harmful behaviours.

The higher rates of stalking among young people are potentially explained by the importance of relationships and the myriad of changes experienced at this developmental stage (Mullen et al., 2009; Zarrett & Eccles, 2006). This means that early education is of the highest importance. However, research by the team on the topic of stalking education has found it to be unequal with a lack of evaluation: only one stalking education programme, the Alice Ruggles Trust Assemblies programme, has been independently evaluated in the United Kingdom ( Scott et al., 2025). The young people we spoke to clearly stated that more education was needed, and they were confident in their ideas about what education should look like and that it should be inclusive. Although greater gender representation and consideration for different learning styles were called for in the workshops in ways that reflect calls in the literature (Bovill et al., 2023), further inclusivity should be addressed in terms of stalking education that appeals to minority groups, who may be at greater risk of experiencing and minimising stalking behaviours (Janickyj et al., 2025). The young people we spoke to also expressed a desire for a varied approaches that include multiple forms of media, in-person and digital approaches, and follow-ups, approaches which are reflected as effective in the literature (Addis & Snowdon, 2023; PSHE Association, 2021; White & Carmody, 2018). There was also an interest in hearing real-life stories, an approach which other Welsh initiatives, such as knife crime prevention, suggest may be effective (South Wales Police, n.d.). Further research into the impact of hearing from those impacted by stalking on young people in and outside of formal education or work is warranted.

Finally, the workshop model developed for the purposes of the study proved to be engaging. As detailed above, an unexpected outcome from the study was that young people reported leaving the workshop more informed. This suggests promise for an interactive, iterative, longer-duration and workshop-style approach to stalking education that does not mimic traditional learning formats. Although this has risks, such as not fitting into traditional learning schedule slots, the widespread concern about stalking among young people demands the development and roll-out of intensive learning opportunities that can provide space for young people to reflect and debate the issue and feel involved in its prevention.

## Limitations

We engaged with a breadth of young people across Wales in a range of formal and informal settings. However, recruitment issues were encountered. Future research would benefit from deeper partnerships with communities, educational institutions and youth organisations. Such partnerships can only be achieved with a longer duration than our one-year project and with greater funding investment to ensure reciprocal relationships, access to larger groups and enable young people to be embedded as project partners from the outset. Furthermore, although we spoke to a range of diverse groups of young people, demographic information such as gender, ethnicity, sexuality and socio-economic background was not collected or measured. This choice was made to reduce any risk of identification, decrease any potential feelings of intrusion or profiling for the young people and reduce the administrative burden on participants already engaged in a three-hour workshop. The research at this stage also aimed to identify shared meanings, perceptions and views, rather than disaggregating based on background or location. However, the existing literature demonstrates that more knowledge is needed about how to prevent stalking among minority groups, for example, ethnic or sexual/gender minority groups where there are complex interrelations between stalking and other crimes such as honour-based abuse, homophobia and overall disadvantages such as discrimination (Bhanbhro, 2023; Rothman et al., 2021; Sheridan et al., 2019). Future research will benefit from gathering this information to look at correlations between background, perceptions and exposure to education. A further limitation concerns the age group. Due to time and budget restrictions, we focused on the age group 16 to 24, but more research is needed with younger groups (Barr & Newman, 2024).

Finally, although this project engaged young people from different parts of Wales to gain a breadth of input and not only prioritise south Wales, it must also be acknowledged that Wales is a complex small nation within which populations experience variables such as a range of intersecting (dis)advantages, differing local policies and varying cultural priorities. For example, this project was not delivered in the medium of Welsh. Future research should consider the influence of geographical, cultural and linguistic influences on perceptions, opinions and language within nations such as Wales, in addition to how other national identities and contexts might influence the stalking perceptions held by young people.
